# Inferences about global scenario of HTLV-1 infection using data mining of viral sequences

**DOI:** 10.1186/1742-4690-11-S1-P48

**Published:** 2014-01-07

**Authors:** Thessika HA Araujo, Fernanda K Barreto, Aline CAM Miranda, Luiz CJ Alcantara

**Affiliations:** 1 Gonçalo Moniz Research Center/Oswaldo Cruz Foundation, Salvador, Bahia, Brazil; 2 Bahia School of Medicine and Public Health/Bahia Foundation for Science Development, Salvador Bahia, Brazil; 3 Institute of Health Sciences, Federal University of Bahia, Salvador, Bahia, Brazil

## 

The human T-lymphotropic virus type 1 (HTLV-1) infects 15 to 20 million individuals throughout the world. Several HTLV-1 viral sequences are stored in HTLV-1 Molecular Epidemiology Database. The objective of this study was to describe the clinical, molecular and epidemiological scenarios of HTLV-1 infection through the stored sequences in the HTLV-1 Molecular Epidemiology Database. We have used the search algorithm implemented at the HTLV-1 database and the Excel to perform the descriptive analyses. There are currently 2,544 registered sequences with 2,057 (80.85%) of those sequences representing unique isolates. Among these sequences, 1,913 (92.99%) contained geographic information and only 797 (38.74%) are related to clinical status (TSP/HAM, 43%, ATLL, 19%, HC, 32.39%, and other diseases, 5.61%). 1,090 sequences contained information about geographic origin and viral subtype. Of these sequences 1,019 represent subtype a. The data about ethnicity are very scarce .Only 40 sequences have this information. 31 of them have information about clinical status, viral subtype, ethnicity and age at the same time. All of them were originated from women and 11 of them were younger than 40 years old. About the clinical status 9 of them were TSP/HAM and 21 HC. The HTLV-1 Molecular Epidemiology Database enabled some inferences about specific aspects on HTLV-1 infection, however due to the relative scarcity of data about the available sequences it was not possible to delineate a global scenario of HTLV-1 infection. Viral sequence data should be more offered because they can be used for planning public health policies.

